# Adults with tetralogy of Fallot show specific features of cerebral small vessel disease: the BACH San Donato study

**DOI:** 10.1007/s11682-022-00629-6

**Published:** 2022-03-09

**Authors:** Luca Melazzini, Filippo Savoldi, Massimo Chessa, Paolo Vitali, Moreno Zanardo, Enrico Giuseppe Bertoldo, Valentina Fiolo, Ludovica Griffanti, Mario Carminati, Alessandro Frigiola, Alessandro Giamberti, Francesco Secchi, Edward Callus, Marina Codari, Francesco Sardanelli

**Affiliations:** 1 Department of Biomedical Sciences for Health, Università degli Studi di Milano, Via Mangiagalli 31, 20133 Milano, Italy; 2 Postgraduate School in Radiodiagnostics, Università degli Studi di Milano, Via Festa del Perdono 7, 20122 Milano, Italy; 3 ACHD Unit, Pediatric and Adult Congenital Heart Centre, IRCCS Policlinico San Donato, Via Morandi 30, 20097 San Donato Milanese, Italy; 4 Vita-Salute San Raffaele University,, Via Olgettina 58, 20132 Milano, Italy; 5 Unit of Radiology, IRCCS Policlinico San Donato, Via Morandi 30, 20097 San Donato Milanese, Italy; 6 Clinical Psychology Service, IRCCS Policlinico San Donato, Via Morandi 30, 20097 San Donato Milanese, Italy; 7 Department of Psychiatry, Wellcome Centre for Integrative Neuroimaging (WIN), University of Oxford, Warneford Ln, Headington, OX3 7JX Oxford UK; 8 Department of Pediatric Cardiology and Adult Congenital Heart Disease, IRCCS Policlinico San Donato, Via Morandi 30, 20097 San Donato Milanese, Italy; 9 Department of Congenital Cardiac Surgery, IRCCS Policlinico San Donato, Via Morandi 30, 20097 San Donato Milanese, Italy; 10 Department of Radiology, School of Medicine, Stanford University, 300 Pasteur Drive, Stanford, CA 94305-5105 USA

**Keywords:** Aging, Congenital heart defects, White matter disease, Brain vascular injury, Neuropsychological tests

## Abstract

Life expectancy in adults with congenital heart disease (ACHD) has increased. As these patients grow older, they experience aging-related diseases more than their healthy peers. To better characterize this field, we launched the multi-disciplinary BACH (Brain Aging in Congenital Heart disease) San Donato study, that aimed at investigating signs of brain injury in ACHD. Twenty-three adults with repaired tetralogy of Fallot and 23 age- and sex-matched healthy controls were prospectively recruited and underwent brain magnetic resonance imaging. White matter hyperintensities (WMHs) were segmented using a machine-learning approach and automatically split into periventricular and deep. Cerebral microbleeds were manually counted. A subset of 14 patients were also assessed with an extensive neuropsychological battery. Age was 41.78 ± 10.33 years (mean ± standard deviation) for patients and 41.48 ± 10.28 years for controls (*p* = 0.921). Albeit not significantly, total brain (*p* = 0.282) and brain tissue volumes (*p* = 0.539 for cerebrospinal fluid, *p* = 0.661 for grey matter, *p* = 0.793 for white matter) were lower in ACHD, while total volume (*p* = 0.283) and sub-classes of WMHs (*p* = 0.386 for periventricular WMHs and *p* = 0.138 for deep WMHs) were higher in ACHD than in controls. Deep WMHs were associated with poorer performance at the frontal assessment battery (r = -0.650, *p* = 0.012). Also, patients had a much larger number of microbleeds than controls (median and interquartile range 5 [3–11] and 0 [0–0] respectively; *p* < 0.001). In this study, adults with tetralogy of Fallot showed specific signs of brain injury, with some clinical implications. Eventually, accurate characterization of brain health using neuroimaging and neuropsychological data would aid in the identification of ACHD patients at risk of cognitive deterioration.

## Introduction

Surgical and medical advancements have allowed people born with congenital heart defects to live longer. Mortality in these patients has shifted away from infancy to adulthood, leading to the development of a large population of adults with congenital heart disease (ACHD) (Diller et al., 2015). Although most infants can go on to lead a normal childhood and adolescence after correction of the heart defect(s), grown-up patients require continuous medical attention and are particularly exposed to acquired cardiovascular disorders (Marelli et al., 2016).

The association between heart failure, a highly prevalent complication in ACHD, and both imaging signs of brain injury (Vogels et al., 2007) and impaired cognitive functioning (Trojano et al., 2003) has been extensively addressed. The risk of brain injury in patients with cardiovascular disorders goes well-beyond the clinical setting of symptomatic heart failure and involves both heart dysrhythmia (Mayasi et al., 2018) and acquired vascular disorders (Friedman et al., 2014). For this reason, the hypothesis of dementia as a cardiovascular-driven disease has been recently put forward (de Roos et al., 2017), supported by the increasing evidence of the often co-existing signs of cerebrovascular disease with findings of Alzheimer’s pathology (Attems & Jellinger, 2014).

People with ACHD are prone to developing acquired cardiovascular risk factor for brain injury and are thereby expected to experience dementia more than the general population (Bagge et al., 2018). Reduced brain reserve due to neurodevelopmental alterations in infancy is the substrate on which acquired cardiovascular disorders act as triggers for impaired cognition (Mebius et al., 2018). Despite the abundance of studies dealing with neurodevelopmental issues in children with heart defects, few studies investigated brain structure and cognitive functioning in ACHD (Marelli et al., 2016; Melazzini et al., 2019).

Moreover, available studies on brain integrity in ACHD carry several limitations such as, among others, inclusion of patients with unrepaired heart defects (Cordina et al., 2014; Horigome et al., 2006; Jensen et al., 2015), heterogeneous study samples as regards type and severity of congenital heart disease (Kessler et al., 2020), lack of control groups (Chai et al., 2018; Jensen et al., 2015; Sluman et al., 2017), undisclosed imaging protocols (Sluman et al., 2017) and qualitative-only characterization of the imaging signs of brain injury (Horigome et al., 2006; Jensen et al., 2015). With the only exception of a brief neuropsychological assessment performed in (Sluman et al., 2017) and an estimate of the intelligence quotient in (Kessler et al., 2020), the available studies did not investigate the associations between patients’ imaging findings and cognitive functioning.

In our previous work, we performed a preliminary study on a homogenous sample of adult patients with repaired tetralogy of Fallot, the most common form of complex congenital heart disease (Apitz et al., 2009), who underwent magnetic resonance imaging (MRI) of the brain to explore the occurrence of signs of cerebrovascular damage. We found an increased prevalence of cerebral microbleeds (CMBs) in the patients’ group compared to their age- and sex-matched healthy peers. The number of CMBs was not associated with neither severity of symptoms of heart failure, age at corrective surgery or use of extracorporeal circulation. However, patients with more severe symptoms of heart failure showed higher white matter hyperintensities (WMHs) volume. The observed imaging findings suggested an increased susceptibility to brain damage and precocious brain ageing in ACHD patients (Codari et al., 2018), which prompted the need for a larger validation and a more in-depth evaluation of the relationship with cognition.

Considering this, in addition to the limited available evidence on the topic of cerebrovascular damage in ACHD, we set out to expand the sample from our preliminary study and launched the BACH (Brain Aging in Congenital Heart disease) San Donato study. We investigated the prevalence of imaging signs of brain vascular damage and their association with patients’ cognitive functioning.

## Materials and methods

### The BACH study population

The BACH study is an observational cross-sectional prospective study that aims at identifying whether ACHD patients show more signs of cerebrovascular damage than their healthy peers.

**Fig. 1:**
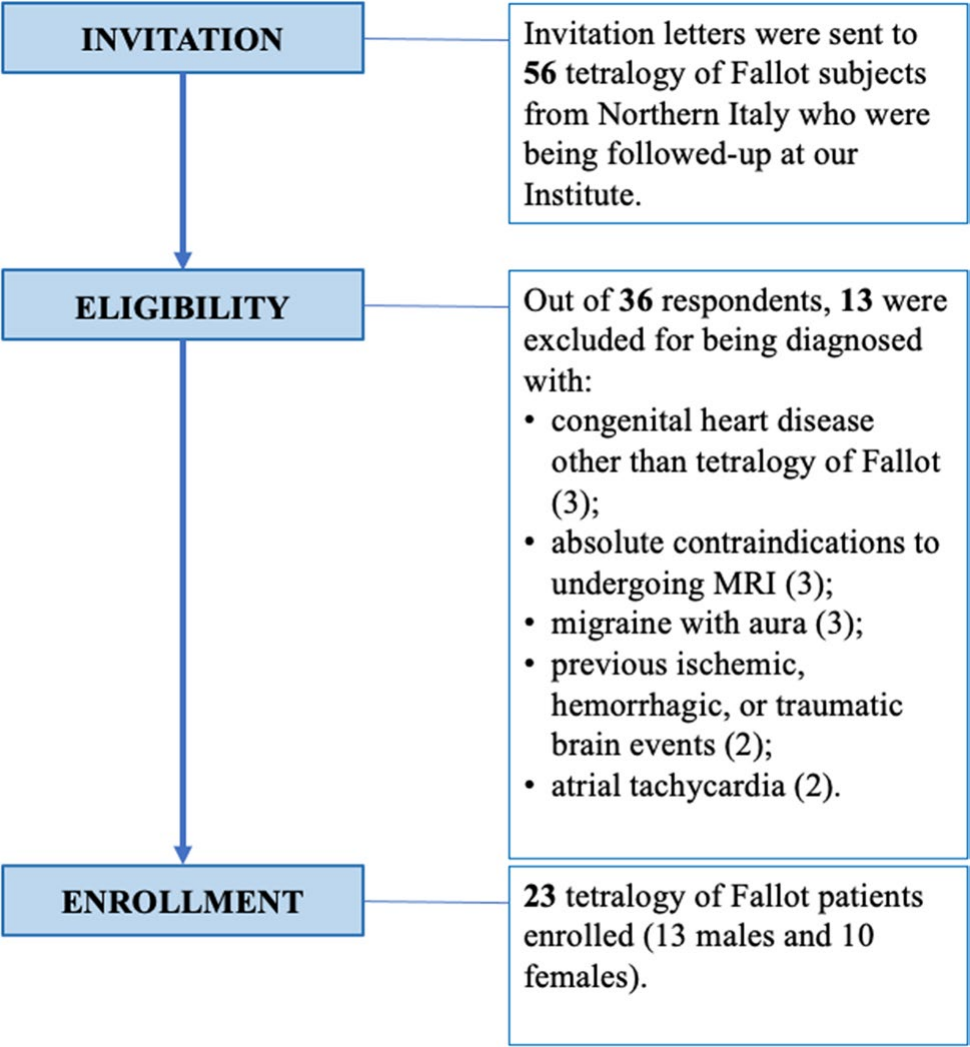
Flowchart depicting patient selection and inclusion/exclusion criteria for the study

To be included in this study, subjects had to be more than 18 years of age and with a tetralogy of Fallot diagnosis, regardless of the type of corrective surgery they underwent. To contact eligible patients, we sent out invitation letters signed by the chief of the radiology, cardiology, cardiac surgery and clinical psychology units of the IRCCS Policlinico San Donato to all tetralogy of Fallot subjects from Northern Italy who were being followed up at our Institute. Once interested patients contacted us, they were reached by phone to check for exclusion criteria. Patients were excluded from the study if they presented any of the following: absolute contraindications to undergoing an MRI scan; pregnancy; inflammatory, infectious, demyelinating or dysmyelinating diseases of the central nervous system; ischemic, haemorrhagic, or traumatic brain events with possible gliotic, malacic, or lacunar sequelae; mendelian or mitochondrial genetic diseases of the central nervous system, including cerebral autosomal dominant arteriopathy with subcortical infarcts and leukoencephalopathy; cerebral amyloid angiopathy; cerebral arteriovenous malformations; primary or metastatic brain neoplasms; previous cranial/brain surgery; patent oval foramen; atrial tachycardia; pregnancy; migraine with aura. Patient selection criteria are depicted in Fig. 1.

The ethical committee of the San Raffaele Research Hospital approved this study on May 10^th^, 2018 (protocol name: LEUCO) and written informed consent was obtained from all enrolled subjects. Upon signing the informed consent, a questionnaire on lifestyle was administered and, for patients only, the most recent cardiological evaluation was also collected. The questionnaire was conceived to include numeric response questions only; data on total education, smoking habits, alcohol intake and physical activity were collected. The New York Heart Association (NYHA) score for symptoms of heart failure, age at corrective surgery, number of cardiac surgeries using extra-corporeal circulation, number of percutaneous cardiac interventions, systolic and diastolic blood pressure and blood oxygen saturation were extracted from the cardiological evaluation.

### Imaging protocol and image processing

Information on the imaging protocol and image processing for the 20 subjects enrolled in the preliminary study was previously described (Codari et al., 2018).

Brain MRI scans were acquired for the 26 new subjects (13 patients and 13 healthy controls) using a 1.5-T scanner (Magnetom Symphony Tim, Siemens Healthineers, Erlangen, Germany) equipped with a 12-channel head coil using the protocol reported in Table 1.

**Table 1:** MRI protocol sequence parameters

MRI Sequence	Inversion Time (ms)	Echo Time (ms)	Repetition Time (ms)	Flip Angle (°)	Resolution (mm^3^)
3D gradient echo (GRE) T1-weighted	900	2.8	2,200	8	0.98 × 0.98 × 1.00
3D turbo spin echo fluid-attenuated inversion recovery	2,200	358	6,000	120	1.02 × 1.02 × 1.00
Axial GRE T2*-weighted	-	26	822	20	0.45 × 0.45 × 4.00

All images were processed and analysed using FMRIB Software Library (FSL) v.6.0 tools (Jenkinson et al., 2012). We used FSL-FAST (Zhang et al., 2001) on T_1_-weighted images to perform tissue type segmentation and calculate maps of partial volume estimates (PVE) for the three brain tissue classes (grey matter [GM], white matter [WM] and cerebrospinal fluid [CSF]). Total volumes for the three tissue classes were obtained by multiplying the PVE output mean by the output volume for non-zero voxels. White matter hyperintensities segmentation was automatically performed with the algorithm FSL-BIANCA on FLAIR images (Griffanti et al., 2016). The algorithm was trained with the WMH masks manually delineated in the preliminary study sample (*n* = 20) (Codari et al., 2018) and applied on the 26 new subjects. The algorithm output underwent subsequent postprocessing to automatically classify WMHs into periventricular and deep (Melazzini et al., 2021a). The segmentation output was manually inspected and, if needed, manual refinement was also performed by a Ph.D. student with 6 years of clinical experience, specifically trained by a neuroradiologist, using ITK-SNAP (Yushkevich et al., 2006). Manual count of CMBs on T2*-weighted images was performed by the same operator who manually corrected the WMH masks.

### Neuropsychological assessment

Patients underwent a thorough neuropsychological examination that investigated attention, executive functioning, memory, and the visuospatial and sensorimotor systems. Notably, the following tests were administered: mini-mental state examination (Giovanni Measso, Cavarzeran, et al., 1993; Measso, Zappalà, et al., 1993), frontal assessment battery (FAB) (Appollonio et al., 2005), symbol digit test (Amodio et al., 2002), attentive matrices (Della Sala et al., 1992), trail-making test A and B (Giovagnoli et al., 1996), Weigl’s sorting test (Inzaghi, 2010; Laiacona et al., 2000), digit span test (Orsini et al., 1987), visuospatial span (Corsi, 1973; Orsini et al., 1987), Babcock story recall text (Carlesimo et al., 2002), letter fluency (Caltagirone et al., 1995; Carlesimo et al., 1995), category fluency (Novelli et al., 1986), pantomime of object use (De Renzi et al., 1980) and coloured progressive matrices (G Measso et al., 1993; Measso, Cavarzeran, et al., 1993). Test raw scores were adjusted for age and education and transformed into equivalent scores (ES) in the form of a 5-point scale (0–4, where 0 indicates a performance lower than the fifth centile of the normal population, and an ES of 4 means equal or better than the median) as described in Capitania and Laiacona (1997). If alternate forms of the same tests were available, they were used in order to limit the possible learning effect of the patients in the re-test phase (Benedict & Zgaljardic, 1998). A brief description of each test is reported in Table 2.

**Table 2:** Synopsis of the administered neuropsychological tests

Test Name	Test Version	Cognitive domain	Description of the test
Mini Mental State Examination (MMSE)	(Giovanni Measso, Cavarzeran, et al., 1993; Measso et al., 1993)	Brief mental status screening	It is composed of 30 items assessing spatial and temporal orientation, memory, attention and calculation abilities, language abilities and praxis
Frontal Assessment Battery (FAB)	(Appollonio et al., 2005)	Global screening of executive functioning	It is a brief battery composed of six subtests assessing conceptualization skills, mental flexibility, programming, sensitivity to interference, inhibitory control and gripping behavior
Symbol Digit Test (SDT)	(Amodio et al., 2002)	Sustained attention and focus	It tests the capability to link a number to a specific symbol in a short time
Attentive matrices	(Della Sala et al., 1992)	Selective attention	It is a tool for assessing visual attention, speed and detection skills, working memory and visual-attentional processes
Trail-making test (TMT) version AB	(Giovagnoli et al., 1996)	Visual attention, visual-motor coordination, ability to alternate the conceptual setting and mental flexibility	The TMT is made up of two subtests A and B. In the first task the patient has to link in sequence all the numbers present in a sheet. In the second task, the patient must alternate between a number and a letter in the shortest possible time
Weigl’s sorting test	(Inzaghi, 2010)	Ability to categorize and cognitive flexibility	The test consists of twelve wooden objects that the patient has to categorize according to the examiner’s sort condition
Digit span test	(Orsini et al., 1987)	Short-term verbal memory span measurement	Oral presentation of a series of figures. If the patient repeats the figures in the same order they are presented, he moves on to the longer series
Visuo-Spatial span (Corsi tapping test)	(Corsi, 1973)	Visual-spatial span measurement	The patient is presented with a tablet with nine cubes. The patient has to reproduce the sequence in the same order
Babcock story recall text	(Carlesimo et al., 2002)	Verbal long-term memory assessment	The story consists of four main elements and five secondary elements. The rehearsal takes place in immediate and deferred recall
Letter fluency	(Caltagirone et al., 1995)	Ability to access the verbal lexicon by the phonological way	It is a test to evaluate lexical access, sensitive to left brain damage and frontal injuries
Category fluency	(Novelli et al., 1986)	Ability to access access to the lexicon by the semantic way	The patient is asked to name the largest number of words belonging to certain categories (e.g. fruits, animals, car brands)
Pantomime of object use	(De Renzi et al., 1980)	Aspects of ideational and ideo-motor praxis of the upper limbs	The patient is asked to perform the pantomime of the use of different objects
Coloured progressive matrices (CPM-47)	(Measso et al., 1993; Measso et al., 1993)	Visual-spatial analogical reasoning and logical-deductive intelligence	The test consists of 36 boards, divided into three series (A, AB, B). Each table consists of an upper part which contains the stimulus figure which lacks a fragment and a lower part which contains six response alternatives

This part of the study was carried out in cooperation with the Clinical Psychology Service at IRCCS Policlinico San Donato. The neuropsychological assessment was conducted by two qualified neuropsychologists (EGB and VF) with high level of experience in the adult and older adult cognitive functioning and assessment. The total time for the administration of the neuropsychological battery was approximately 60–75 min.

### Statistical analysis

After checking for normality of data distribution using the Shapiro–Wilk test, demographic variables in the two groups were compared using either the *t*-test or the Mann–Whitney *U* test. Significant differences on brain tissue volumes and WMH volumes between ACHD and healthy controls were explored using the analysis of covariance (ANCOVA) approach, using the total brain volume as covariate for adjusting for individual differences in brain size (O’Brien et al., 2011). *P*-values in ANCOVA were corrected for multiple comparisons using the Bonferroni method (Bonferroni, 1936). Pearson’s correlation coefficient (*r*) was used for quantifying the associations between MRI volumetric data and demographics, clinical data, or neuropsychological tests scores. The Mann–Whitney *U* test was used to compare CMBs between ACHD patients and healthy controls, and the nonparametric Spearman’s correlation coefficient (ρ) was used for testing any associations between CMBs count and volumetric data, demographics, clinical data, or neuropsychological tests scores. Statistical significance level was set at *p*-value ≤ 0.05 (Di Leo & Sardanelli, 2020).

Statistical analysis was performed using SPSS version 25.0 (IBM Corp., Armonk, NY).

## Results

The patients’ group consisted of 13 males and 10 females (n = 23), with a mean (standard deviation [SD]) age of 41.78 (10.33) years. The controls’ group was composed of age- and sex-matched healthy subjects (n = 23), with a mean (SD) age of 41.48 (10.28) years. Demographics related to the patients’ and healthy controls’ groups and clinical data for ACHD patients are summarised in Table 3.

**Table 3:** Demographics and clinical data of the study sample. Both the patients’ and healthy controls’ groups comprised 13 male and 10 female participants each (*P*-value = 1). Clinical data are available for the patients’ group only

	ACHD patients (n = 23)				Healthy controls (n = 23)				*P*-values^#^
*Demographics*	Mean	SD	Median	IQR	Mean	SD	Median	IQR	
Age [years]	41.78	10.33	46.00	33.00—49.00	41.48	10.28	46.00	32.50—48.50	0.851
Education [years]	14.76	4.10	16.00	13.00—17.00	14.13	3.28	16.00	13.00—16.00	0.631
Body mass index [kg/m^2^]	24.41	4.07	24.88	21.00—27.31	24.97	3.93	24.39	22.75—26.77	0.642
Smoking [pack/day*year]	0.02	0.11	0.00	0.00—0.00	3.83	11.50	0.00	0.00—0.00	0.084
Alcohol intake [units/week]	3.59	6.87	1.00	0.00—3.50	4.83	5.01	4.00	1.00—7.00	0.122
Physical activity [h/week]	2.41	2.68	2.00	0.00—3.75	1.52	1.31	1.00	0.00—3.00	0.485
*Clinical data (patients only)*									
NYHA [score I-IV]	1.57	0.59	2.00	1.00—2.00					
Age at corrective surgery [months]	50.91	0.59	48.00	24.00—63.00					
Cardiac surgeries using ECC	1.78	0.67	2.00	1.00—2.00					
Percutaneous interventions	0.26	0.62	0.00	0.00—0.00					
SBP [mmHg]	117.31	12.51	119.50	105.00—130.00					
DBP [mmHg]	71.38	12.51	72.50	63.00—80.00					
Sat0_2_ [%]	96.88%	5.68%	98%	97—99%					

*ACHD*: adults with congenital heart disease; *SD*: standard deviation; *NYHA*: New York Heart Association scale for symptoms of heart failure; *ECC*: extracorporeal circulation; *SBP*: systolic blood pressure; *DBP*: diastolic blood pressure; *Sat0*_*2*_: fraction of oxygen-saturated haemoglobin relative to total haemoglobin

^#^Distribution of variables was analysed using the Shapiro–Wilk test. Age and body mass index were compared using the t-test due to symmetrical distribution of data. Education, smoking, alcohol intake and physical activity were compared using the Mann–Whitney *U* test due to asymmetrical distribution of data

We did not find any statistically significant difference in demographics and clinical data distributions between ACHD patients and healthy controls.

Median and interquartile range (IQR) for number of CMBs in the patients’ group were 5 [3–11] CMBs and 0 [0–0] CMBs in the healthy controls’ group (*p* < 0.001). Cerebral microbleeds were found in 22 (95.7%) out of 23 patients and in one (4.3%) out of 23 healthy controls.

Differences in brain imaging volumetric data between patients and healthy controls did not reach statistical significance.

Mean and SD for volumetric brain imaging measures (WMHs, WMH sub-classes and brain tissues volumes) are reported in Table 4.

**Table 4:** Brain imaging volumetric data of the study sample

	ACHD patients (n = 23)				Healhy controls (n = 23)				*P*-values^#^
	Mean	SD	Median	IQR	Mean	SD	Median	IQR	
WMHs [ml]	3.99	2.07	3.15	2.47—5.43	3.57	1.71	3.50	0.97—4.21	0.283
WMHs Periventricular [ml]	3.56	1.74	3.09	2.35—4.88	3.32	1.55	3.36	0.96—3.90	0.386
WMHs Deep [ml]	0.43	0.52	0.29	0.086—0.55	0.25	0.23	0.19	0.01—0.35	0.138
CSF [ml]	264.30	30.99	257.30	239.20—282.91	275.04	31.66	279.48	213.56—289.93	0.539
GM [ml]	561.41	49.78	561.67	517.71—584.33	572.08	47.17	585.01	459.70—598.88	0.661
WM [ml]	510.37	52.28	495.59	470.96—542.92	526.18	63.28	519.13	376.25—584.67	0.793
TBV [ml]	1,336.08	109.06	1,313.17	1,258.58—1,414.19	1,373.30	122.50	1,401.70	1,064.77—1,477.31	0.282

*ACHD*: adults with congenital heart disease; *SD*: standard deviation; *IQR*: interquartile range; *WMHs*: white matter hyperintensities; *CSF*: cerebrospinal fluid; *GM*: grey matter; *WM*: white matter; *TBV*: total brain volume (i.e., sum of CSF, GM and WM)

^#^*P*-values were obtained from analysis of covariance (ANCOVA) with total brain volume as covariate

The neuropsychological test battery was introduced following the preliminary results described in (Codari et al., 2018) and was thereby performed on 14 out of 23 (60.87%) ACHD patients. Mean and SD of neuropsychological tests equivalent scores are reported in Table 5.

**Table 5:** Overview of neuropsychological tests scores. A higher value indicates a better performance (equivalent scores, range 0–4)

	Mean	Standard deviation	Median	Interquartile range
Mini-Mental State Examination	4.00	0.00	4.00	4.00 – 4.00
Frontal assessment battery	2.86	1.29	3.00	2.00 – 4.00
Symbol Digit Test	4.00	0.00	4.00	4.00 – 4.00
Attentive matrices	3.14	0.86	3.00	2.25 – 4.00
Trail making test A-B	3.57	1.16	4.00	4.00 – 4.00
Weigl’s sorting test	3.21	0.70	3.00	3.00 – 4.00
Digit span test	3.07	1.44	4.00	2.25 – 4.00
Babcock story recall text	3.14	1.10	3.50	3.00 – 4.00
Letter fluency	3.64	0.63	4.00	3.25 – 4.00
Category fluency	3.79	0.58	4.00	4.00 – 4.00
Pantomime of object use	4.00	0.00	4.00	4.00 – 4.00
Coloured Progressive Matrices	3.57	0.76	4.00	3.25 – 4.00

**Fig. 2:**
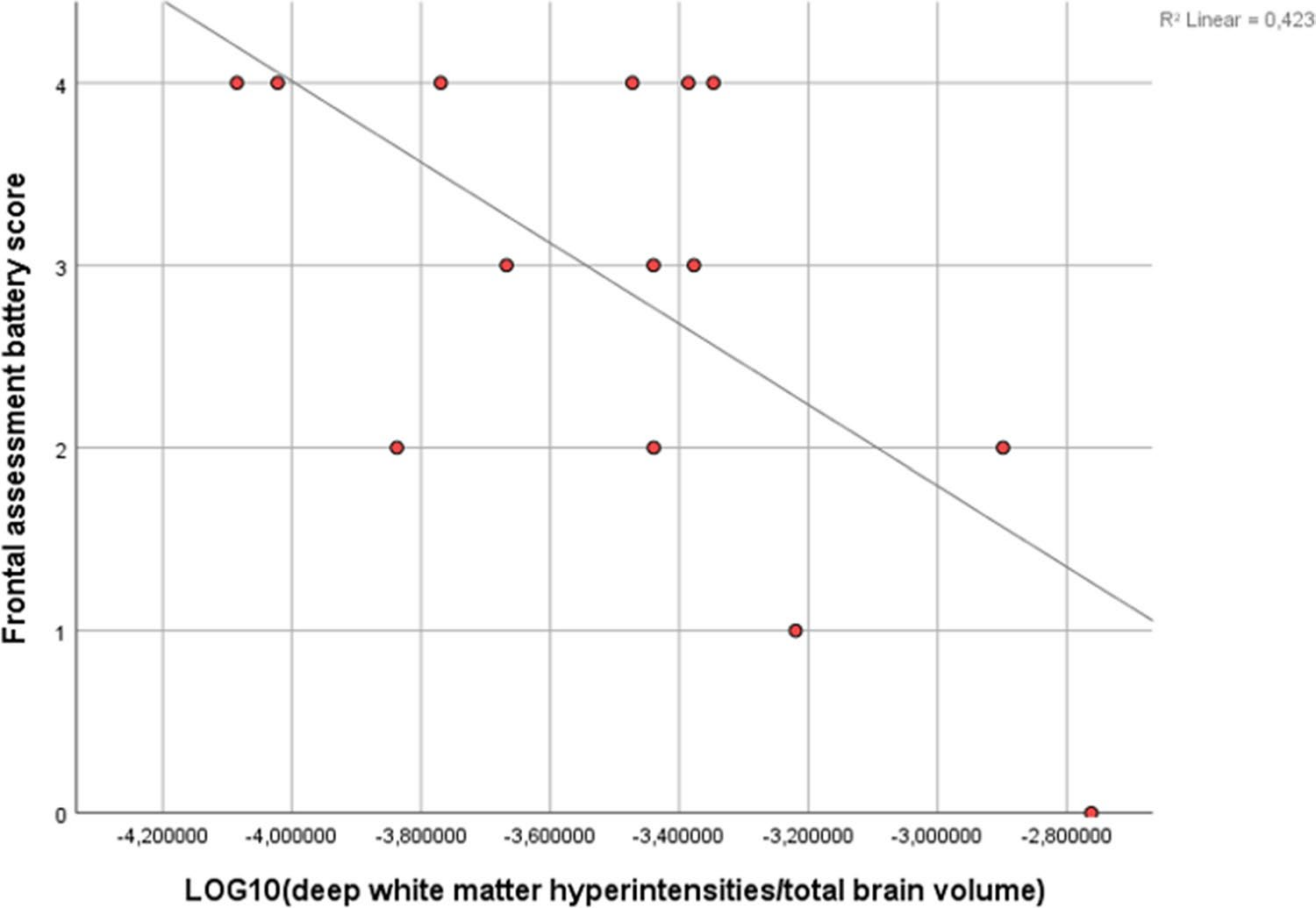
Scatter diagram with linear regression line for frontal assessment battery score on normalised deep white matter hyperintensities volume

Higher deep WMH volume was significantly associated with poorer FAB scores (*r* = -0.650, *p* = 0.012; Fig. 2). Among other volumetric data, a higher GM volume was significantly associated with better scores at the coloured progressive matrices test (*r* = 0.598, *p* < 0.001). Cerebral microbleeds were not associated with the cognitive performance in any of the neuropsychological tests.

We did not find any statistically significant association between volumetric imaging data and demographics, clinical data or cognitive performance. Cerebral microbleeds were positively associated with the amount of physical activity per week (*r* = 0.329, *p* = 0.027).

## Discussion

The aim of this study was to investigate signs of cerebrovascular damage in a group of adult patients with tetralogy of Fallot. Coherently with our preliminary findings (Codari et al., 2018), we found a considerably higher number of cerebral microbleeds in ACHD patients than in controls. Albeit not significant, WMH total volume and subclasses of WMHs were higher, while total brain and brain tissues volumes were lower, in ACHD patients than in controls. Considering that all these measures are in favour of a relatively injured brain in ACHD patients, it is likely that the lack of statistically significant differences may be due to the relatively small sample size. Interestingly, we found significantly higher deep WMH volume in patients who performed worse at the FAB.

Cerebral microbleeds are radiological signs of small vessel disease (SVD) that are visible in T_2_*-weighted and susceptibility-weighted images (Haller et al., 2018). They are commonly associated with hypertension and cerebral amyloid angiopathy and are linked to an increased risk of all-type dementia in patients with vascular risk factors (Miwa et al., 2014). It has been found that 76% of patients performing cardiac surgery showed cerebral microbleeds that were absent before the heart intervention (Patel et al., 2019). It is worth noting that CMBs were detected in 96% of our patients; this very high prevalence may suggest an intrinsic influence of the heart disease on developing CMBs, independently from any cardiac procedure. We do not have brain MRI studies available before surgical or percutaneous treatments allowing us to confirm this hypothesis. In this view, serial brain MRI scans before/after interventions may enable the assessment of CMBs over time and characterize their link to cognitive performance. The general meaning of the finding of higher cerebral microbleeds in patients who performed more physical activity is yet to be elucidated.

White matter hyperintensities are radiological signs of SVD and brain frailty (Group, 2015; Melazzini et al., 2021b) and are associated with a twofold increased risk of dementia and a threefold increased risk of stroke (Debette & Markus, 2010). Moreover, WMHs are linked to a faster decline in global cognitive performance, executive function and processing speed (Debette & Markus, 2010). Beside assessing total WMH volume, we sub-classified WMHs into periventricular and deep, in view of our recent finding of sub-classes of WMHs that are specifically linked to poorer cognitive performance in the general population (Melazzini et al., 2021a). In this study, we found that ACHD patients had higher total, periventricular and deep WMH volumes than controls, without reaching statistical significance. However, we excluded patients with acquired disorders that are highly prevalent in ACHD, such as atrial dysrhythmia (Ávila et al., 2017) and migraine with aura (Truong et al., 2008), and that are known to be associated with an increased WMH burden (Bashir et al., 2013; Gaita et al., 2013). For this reason, we believe that the estimate of WMHs in our sample may underestimate the actual volume of total/sub-classes of WMHs in the general tetralogy of Fallot population. Interestingly, we found that higher deep WMH volume in ACHD patients was significantly associated with lower scores at the FAB. This mirrors the finding of lower executive performance with higher deep WMHs in frontal areas, recently described by (Brugulat-Serrat et al., 2019) in a middle-age cohort of cognitively healthy subjects. Sub-classifying WMH proved to be useful despite the small sample size; we thereby believe it may provide added value when investigating the WMHs’ link to cognition.

Total brain and brain tissue volumes were lower in ACHD patients than in controls, albeit not significantly. This finding is coherent with a recent study by (Naef et al., 2021) that found lower total brain volumes in a heterogeneous population of ACHD patients. Higher grey matter volume in our study was significantly associated with better scores at the coloured progressive matrices test. Coloured progressive matrices reasoning ability seems to be related to the activation of networks in the dorsolateral prefrontal cortex (associated with visuospatial abilities) and in the inferior frontal regions (associated with verbal reasoning) (Yang et al., 2014). We did not provide any information about regional grey matter volume in our sample. For this reason, we cannot ascribe this neuropsychological finding to patients’ volumetric data in specific cortical or deep grey matter regions. Segmentation of brain lobes or voxel-based morphometry may give further insights on regional grey matter loss in ACHD patients.

The main limitation of this study is its small sample size. This is essentially due to two reasons. Firstly, we adopted strict inclusion/exclusion criteria on an already-rare population. Secondly, we were not allowed to bring ACHD patients to the hospital for research purposes, since they are at higher risk for severe illness from coronavirus disease. Another limitation is that the neuropsychological test battery was only administered to the ACHD patients, therefore we are currently unable to test if the observed association between deep WMHs and impaired performance at the FAB is disease-specific or could also be found in healthy controls. Also, this study has a prospective cross-sectional design. To establish whether biomarkers of SVD may serve as predictors of cognitive decline in tetralogy of Fallot, patients should be followed up over time and multiple imaging and neuropsychological examinations be performed. Patients from several cardiovascular centres could also be recruited so to avoid recruitment bias. The enrolment of a larger longitudinal multi-centric patient cohort who undertake both brain MRI and cognitive assessment would allow for investigating the reliability, validity, causation, and diagnostic power of the identified brain MRI features. Thereafter, a multi-variate model for the risk of cognitive decline in tetralogy of Fallot could eventually be built and fully translated into clinical practice. Moreover, future studies performing both pre- and post-surgery/catheterization MRI scans could determine how specific cardiac procedures affect brain health.

## Conclusions

Adults with repaired tetralogy of Fallot showed an increased burden of brain vascular damage. We believe that our findings may help improve the understanding on the implications that congenital heart diseases carry on the cerebrovascular system. We hope that confirmatory results will come out once the estimated sample size will be reached. Overall, we stress the need for a holistic evaluation of ACHD patients’ health, with the final aim to increase patients’ self-awareness and improve their quality of life.

## Data Availability

The de-identified data are available from the corresponding author upon reasonable request.

## Abbreviations

ACHD: Adults with congenital heart disease
CMB: Cerebral microbleed
CSF: Cerebrospinal fluid
FAB: Frontal assessment battery
FLAIR: Fluid-attenuated inversion recovery
GM: Grey matter
MRI: Magnetic resonance imaging
NYHA: New York Heart Association
IQR: Interquartile range
PVE: Partial volume estimates
SD: Standard deviation
SVD: Small vessel disease
WM: White matter
WMH: White matter hyperintensity

## Declarations

### Reporting guidelines

The Strengthening the Reporting of Observational Studies in Epidemiology - STROBE checklist has been applied.
